# Audiovestibular outcomes in adult patients with cogan syndrome: a systematic review

**DOI:** 10.1007/s00405-024-08878-5

**Published:** 2024-08-07

**Authors:** Alejandro R. Marrero-Gonzalez, Celine Ward, Shaun A. Nguyen, Seth S. Jeong, Habib G. Rizk

**Affiliations:** 1 Department of Otolaryngology-Head and Neck Surgery, Medical University of South Carolina, 135 Rutledge Avenue, MSC550, Charleston, SC 29425 USA; 2 Department of Rheumatology, Medical University of South Carolina, Charleston, SC USA; 3 Department of Otolaryngology and Communication Sciences, SUNY Upstate Medical University, Syracuse, NY USA

**Keywords:** Cogan syndrome, Steroid resistance, Audiovestibular, DMARDs

## Abstract

**Purpose:**

To determine factors associated with steroid responsiveness and efficacy of biologic disease-modifying anti-rheumatic (DMARD) use in patients with Cogan Syndrome (CS).

**Methods:**

A systematic search of Cochrane Library, PubMed, CINAHL, and Scopus was conducted following the Preferred Reporting Items for Systematic Reviews and Meta-analyses guidelines. Any study describing audiometric or vestibular data and pharmacologic treatment in patients with CS was included. Due to limited literature, only case reports/case series were included.

**Results:**

Seventy case reports or case series studies comprising 79 individual cases of CS were included. A difference in vestibular symptoms with a higher prevalence in the steroid-resistant group than the steroid-responsive group was found (79.5% vs 57.9%, p = 0.04). Eighteen (60.0%) patients treated only with oral steroids had no audiological improvement, while twelve (n = 12; 85.7%) patients treated with biologic DMARD showed audiological improvement. The steroid-responsive group had an overall better response to DMARDs than the steroid-resistant group (62.1% vs 45.0%; 100.0% vs 77.8%).

**Conclusions:**

Our study synthesized the available literature to better characterize steroid resistance in patients with Cogan syndrome and treatment outcomes. Vestibular symptoms were noted to be more prevalent in patients who were eventually labeled as steroid resistant. There were higher rates of audiological improvement in patients given biologic DMARDs rather than conventional DMARDs or steroids only. Further studies are needed to characterize each individual vestibular symptom and investigate the utility and timing of biologic DMARDs in managing patients with Cogan syndrome.

**Supplementary Information:**

The online version contains supplementary material available at 10.1007/s00405-024-08878-5.

## Introduction

Cogan’s syndrome (CS) is a rare chronic inflammatory disorder characterized by ocular and vestibular symptoms. CS was first described in 1945 by David Cogan as the cause of nonsyphilitic interstitial keratitis [1]. In 1980, Haynes et al. suggested that the definition of CS could be expanded to include ocular symptoms other than interstitial keratitis or vestibular symptoms different from Meniere-like episodes [2]. Thus, he proposed diagnostic criteria to differentiate between typical and atypical CS. In typical CS, patients present with interstitial keratitis and Meniere-like episodes consisting of hearing loss, vertigo, and tinnitus. The atypical form is usually characterized by a higher degree of association with systemic vasculitis [2]. However, in the face of a chronic disease like CS that often relapses, the distinction between typical and atypical forms has become less clear and, as a result, less critical [3, 4]. The diagnosis is now based on audiovestibular symptoms, ocular inflammation, nonreactive serological tests for syphilis, and laboratory findings [5].

In the management of CS, oral or systemic corticosteroids remain the standard of care, and high doses can be quite effective in improving auditory symptoms when given within two weeks of initial symptoms [2, 6]. However, corticosteroids are not viable long-term therapies with significant potential complications in long-term use: osteoporosis, joint necrosis, adrenal insufficiency, hyperlipidemia, etc. [7] When a patient is unresponsive, intolerant of steroids, has a specific contraindication, or requires repeated steroid treatments, other immunosuppressive therapies are utilized, such as disease-modifying anti-rheumatic drugs (DMARDs), also called steroid-sparing agents. Patients are considered steroid-resistant if there is no symptomatic improvement within 2 weeks of initiating high-dose corticosteroids at a dose of 0.5–2.0 mg/kg/day. [6, 8, 9]

Conventional DMARD therapies such as cyclophosphamide, methotrexate, cyclosporine, mycophenolate mofetil, and azathioprine have had varying success [10, 11]. However, biologic DMARDs in the form of monoclonal antibodies, such as infliximab, a monoclonal antibody directed against TNFα, have shown promising results [12]. While other biologic DMARDs like tocilizumab (anti-IL6) and rituximab (anti-CD20) have had more limited reported use in CS patients, current studies indicate their potential use for treating symptoms of hearing loss [10, 13]. The aim of this systematic review of case reports/series is to determine (1) factors associated with steroid responsiveness and (2) the efficacy and timing of DMARD use in patients with CS.

## Materials and methods

### Information sources and search strategy

Given the need for more data granularity in the very limited high-quality evidence on Cogan syndrome, we chose to perform a systematic review of case reports. The study was performed according to the Preferred Reporting Items for Systematic Reviews and Meta-Analyses (PRISMA) statement [20]. A comprehensive search was performed in the following 4 databases: PubMed (U.S. national Library of Medicine, National Institutes of Health), Scopus (Elsevier), Cochrane Library (Wiley), and CINAHL (Ebsco). The search strategies used a combination of subject headings (e.g., MeSH in PubMed) and keywords for the following concepts and/or keywords: *Cogan syndrome, audiovestibular symptoms, vestibular symptoms, hearing loss, audiometry, audiometric testing, vestibular function test.* The PubMed search strategy was modified for the other 3 databases, replacing MeSH terms with appropriate subject headings when available and maintaining similar keywords. The search strategies for each database are detailed in **Appendix A**. The databases were searched from inception through August 1, 2023, without filters or limits.

### Study selection

Due to the lack of retrospective and prospective studies, only case reports/series were included. We used a PICOTS (participant, independent variable, comparator or study design, outcome/measure, timing, and setting) framework to structure the eligibility criteria (Supplemental Table 1). Case reports/ series were only included if adult patients were diagnosed with Cogan syndrome based on vestibular symptoms, audiological symptoms, ocular inflammation, nonreactive serological tests for syphilis, and laboratory findings [5]. Furthermore, only studies with audiometric and vestibular data and pharmacologic treatment were included. Editorials/commentaries that did not provide adequate data, comorbidities that complicated Cogan syndrome presentation, nonhuman studies, and review articles were excluded. Lastly, articles with duplicate data from another study were included only once, with the most detailed study included. To identify additional articles, the reference lists of relevant articles and citing articles were hand-searched.

References were exported into the review management software Covidence (Veritas Health Innovation, Melbourne, Australia), for study selection. Two reviewers (S.S.J, A.M.G) independently screened all titles and abstracts. When a disagreement occurred, the relevant articles were discussed between the reviewers until a consensus was reached. Following the same process, two reviewers (S.S.J, A.M.G) then independently screened full-text articles with conflicts being resolved by way of discussion. Two reviewers (S.S.J., A.M.G) searched the reference lists of the included publications to identify additional articles.

### Dealing with heterogeneity

We acknowledge that including case reports introduces inherent heterogeneity in study design, patient characteristics, and reporting standards. Case reports vary in data completeness, quality, and methodological rigor. To address this heterogeneity, we employed rigorous methodology during the data extraction process, emphasizing transparency and consistency. In addition, we appraised the quality and risk of bias in every article. Given the rarity of Cogan’s syndrome, case reports and case series constitute a significant portion of the available literature. Despite the challenges posed by heterogeneity, including case reports and case series allowed us to capture valuable clinical experiences and insights that might not have been otherwise accessible, contributing to a comprehensive understanding of this disease.

### Quality and risk of bias assessment

Articles were critically appraised to assess methodological quality and bias using The Joanna Briggs Institute Critical Appraisal tools for case reports [14]. Two authors (S.S.J, A.M.G) performed a pilot assessment on 5 studies to check for consistency of assessment. Both then performed independent risk assessments on the remaining studies. All disagreements were resolved once both authors came to a consensus. The critical appraisal checklist included a clear description of demographic characteristics, clinical history and timeline, clinical condition on presentation, diagnostic tests or methods and results, intervention or treatment procedure(s), post-interventional clinical condition, adverse events/ unanticipated events, and takeaway lessons. Each item was marked as yes, no, or NA/unclear. (Supplemental Table 2).

### Data collection process and data items

Data extracted from studies included author, publication year, patient demographics (i.e., age, sex), ocular and vestibular symptoms, labs and imaging, duration of symptoms before admission and initiation of treatment, and treatment dose and route of administration. Various outcome data that were extracted included audiometric data (e.g., PTA), vestibular function test (e.g., caloric testing), and response to treatment.

For our study, patients who have symptomatic and audiologic improvement, with a recovery (≥ 15 dB) which is the threshold for clinically significance [15], within 2 weeks of steroid administration, were steroid responsive. The absence of objective audiologic improvement within 2 weeks of steroid administration was considered steroid-resistant. Patients with overall improvement in symptoms but no audiologic recovery were still considered steroid-resistant.

Conventional DMARDs reported in the literature included azathioprine, cyclosporine, cyclophosphamide, leflunomide, methotrexate, and mycophenolate mofetil. Biologic DMARDs included adalimumab (anti-TNFα), infliximab (anti-TNFα), rituximab (anti-CD20), and tocilizumab (anti-IL6). All patients who received conventional or biologic DMARDs also initially received steroids.

Audiological and vestibular outcomes were categorized based on both subjective and audiometric findings into three categories: no improvement, improvement, and resolution. Furthermore, the clinical characteristic of dizziness was collected for articles that described patients with dizziness-like symptoms but no further specific descriptions, such as vertigo or imbalance. Because of this lack of characterization dizziness, vertigo and imbalance were grouped together as vestibular symptoms. Pure-tone averages (PTA) were calculated based on 500 Hz, 1000 Hz, 2000 Hz, and 4000 Hz frequencies. Antibody tests included ANA, ANCA, anti-phospholipids, complements, and cryoglobulins.

### Statistical analysis and synthesis of results

All analyses were conducted using version 28.0 of SPSS (IBM Corporation, Armonk, NY). All continuous variables were tested for normality using Kolmogorov–Smirnov test. Categorical variables were summarized by frequency (N) and percentage (%). Continuous variables were summarized by mean (standard deviation) or median (25th and 75th interquartile range) if the normality test failed. Comparisons of risk factors and outcomes (categorical) were expressed as a percentage with their 95% CI’s and weighted according to the modified Wald method.

Due to the heterogeneity and limitations of the studies included, we have decided not to conduct a quantitative meta-analysis. A traditional meta-analysis, which involves pooling quantitative data from comparable studies, presents unique challenges due to the need for large-scale randomized controlled trials or prospective/retrospective studies. Cogan’s syndrome is a rare disorder, and the number of cases documented in the literature is limited. Therefore, traditional meta-analysis techniques, which require enough homogenous studies, may not be feasible or methodologically appropriate for synthesizing evidence. Instead, we use descriptive and qualitative analysis techniques to provide a narrative synthesis of the evidence. This allows us to explore the breadth and depth of the available information and identify common themes and trends.

## Results

### Search results and study characteristics

**Fig. 1:**
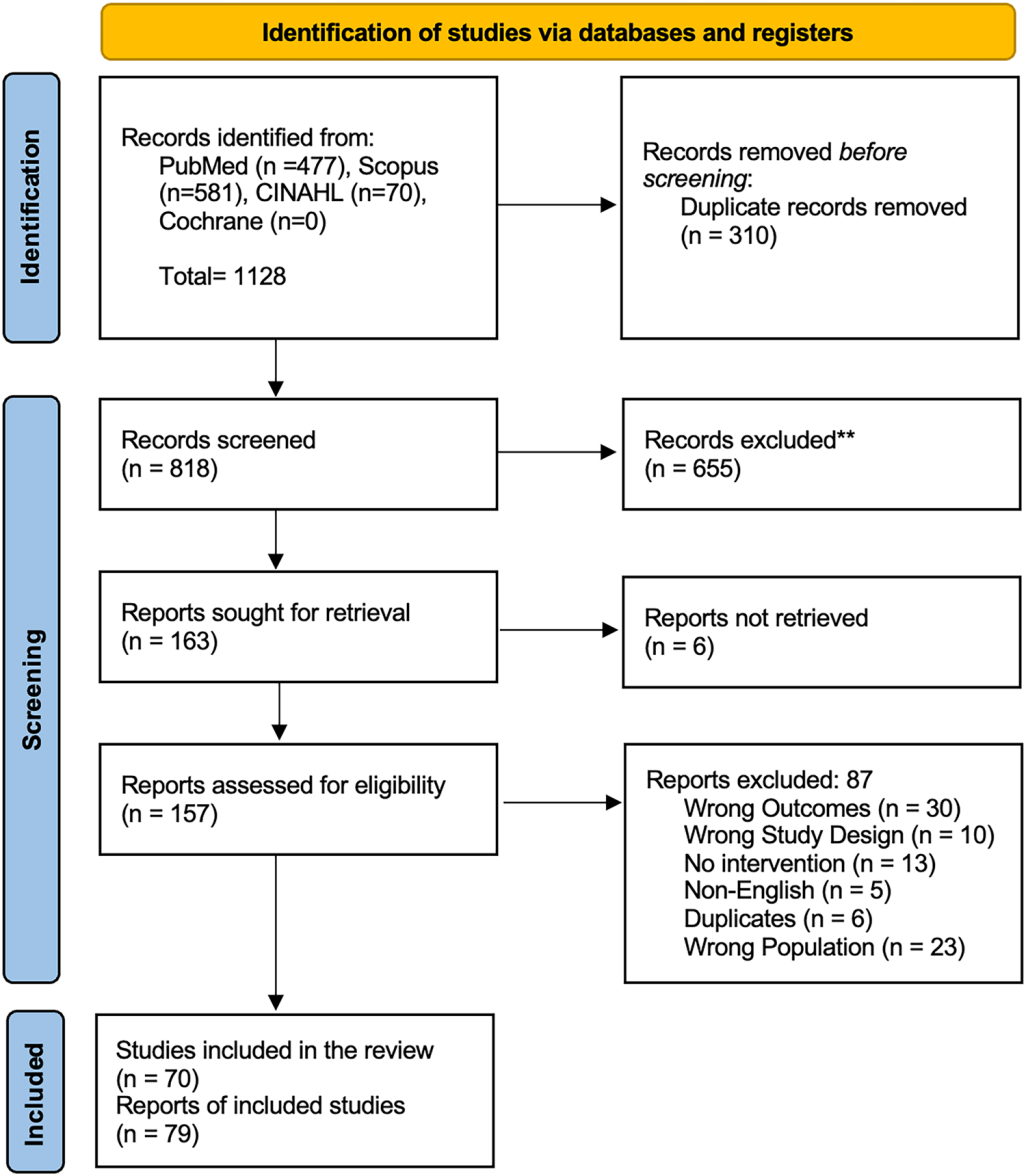
Prisma diagram

The initial literature search identified 1128 articles, with 818 remaining following duplicate removal. Initial title and abstract screening eliminated 655 articles, leaving 157 articles for full-text review. 70 case reports/series were included for final analysis (Fig. 1).

### Descriptive characteristics

The 70 studies (Supplemental Table 3) included a total of 79 individual cases of Cogan syndrome (Table 1). The median age (range) was 33.5 (18.0–82.0) years, and 51.8% (41/78) were female. Of the 79 cases, 60 (75.9%) did not report race. Patients experienced vestibular symptoms for a median (25th–75th IQR) duration of 1.3 months (0.4–8.0) before hospital admission. Thirty-five (44.3%) patients had systemic symptoms, which included fever, arthralgia, gastrointestinal symptoms, neurologic symptoms, etc. Interstitial keratitis (n = 29; 36.7%) was the most frequently documented ocular symptom. The most common otologic symptoms were subjective hearing loss (n = 74; 93.7%), vertigo (n = 38; 48.1%), and tinnitus (n = 44; 55.6%). The least common symptoms were imbalance (n = 22; 27.8%) and dizziness (n = 14; 17.7%). The most common vestibular symptom was vertigo (n = 37; 48.1%). About a third of patients underwent vestibular testing with 22/23 having diminished or absent caloric responses. Of the studies that reported patients with nystagmus, 83.3% reported spontaneous nystagmus. The most common initial symptoms at presentation was hearing loss (n = 58; 73.4%) and vertigo (n = 31; 39.2%). All lab values were found to be outside of the normal reference ranges. For instance, the mean (SD) ESR value was 69.5 mm/hr (34.8), which is much higher than the normal reference range of 0-29 mm/hr. About half the patients with antibody testing, which included qualitative ANA, ANCA, anti-phospholipids, complements, and cryoglobulins, had positive results (n = 23; 46.0%). Head imaging was reported as normal in 28 (66.7%) patients. 14 studies reported abnormal head imaging findings and the most common MRI finding was evidence of labyrinthitis with other findings being encephalitis, neuritis of cranial nerve II – VI and other nonspecific incidental findings. Sensorineural hearing loss was documented following audiological evaluation in all patients. Pooled mean (SD) PTAs (500-4 kHz) at baseline for right and left ears were 67.3 dB (26.8) and 67.7 dB (27.2). Forty-five (56.9%) patients were treated with a conventional DMARD, while 14 (17.7%) patients were treated with biologic DMARD. Before DMARDs were started, all patients had received an initial treatment with steroids. Absent stapedial reflex was reported in 4 (80.0%) patients. The mean duration of steroid treatment (n = 11), regardless of concurrent DMARD therapy, was 1.90 months (1.94). Audiological and vestibular symptom improvement was reported in 36 (45.5%) and 14 (17.7%) patients, respectively.

**Table 1:** Patient characteristics (N = 79)

	Median (25th–75th IQR)	N (%)
*Demographic*		
Age (years)	33.5 (27.3–51.0)	
*Sex*		
Male	–	38 (48.1)
Female	–	41 (51.8)
*Race*		
Caucasian	–	10 (12.6)
African American/Black	–	2 (2.5)
Asian	–	7 (8.9)
Not reported	–	60 (75.9)
*Clinical Characteristics*		
Duration of vestibular symptom before admissions	1.3 (0.4–8.0)	
Presence of systemic symptoms	–	35 (44.3)
*Eye symptoms*		
Keratitis	–	29 (36.7)
Episcleritis/scleritis	–	13 (16.5)
Uveitis	–	6 (7.6)
Other	–	24 (30.4)
Tinnitus	–	44 (55.6)
*Subjective hearing loss*		
Unilateral	–	11 (13.9)
Bilateral	–	63 (79.7)
*Vestibular symptoms*		
Imbalance	–	22 (27.8)
Dizziness	–	14 (17.7)
Vertigo	–	38 (48.1)
*Initial presenting symptoms*		
Hearing loss		58 (73.4)
Dizziness		10 (12.8)
Vertigo		31 (39.2)
Imbalance		17 (21.5)
Ocular Manifestations		30 (37.9)

Lab/findings	Mean (SD)	N (%)
Erythrocyte sedimentation rate (ESR)	69.5 (34.8)	–
C-reactive protein (mg/dL)	8.43 (7.7)	–
Leukocyte count (/mm^3^)	13.0 k (3.6 k)	–
Hemoglobin (g/dL)	10.1 (2.4)	–
Platelets (/mm^3^)	391.4 k (199.0 k)	–
Positive autoimmune test		23/50 (46.0)
Abnormal head imaging		14/42 (33.3)

Audiovestibular test	Mean (SD)	N (%)
Abnormal caloric test		
Normal		1/23 (4.3)
Diminished	–	7/23 (30.4)
Absent	–	15/23 (65.2)
Nystagmus		
Spontaneous	–	5/23 (21.7)
Positional	–	1/23 (4.3)
Not Reported		17/23 (73.9)
Abnormal brainstem auditory evoked response	–	4/23 (17.4)
Absent stapedial reflex	–	4/23 (17.4)
Sensorineural hearing loss		
Unilateral	–	8/59 (13.6)
Bilateral	–	51/59 (86.4)
Right mean PTA (500-4 kHz)	67.3 (26.8)	-
Left mean PTA (500-4 kHz)	69.3 (28.4)	-

Treatment/outcome Characteristics	Median (25th–75th IQR)	N (%)
Time to treatment (months)	1.0 (0.25–2.0)	
Steroid only treatment	–	30 (38.0)
Conventional DMARD treatment	–	45 (56.9)
Azathioprine	–	11 (13.9)
Cyclosporine	–	6 (7.6)
Cyclophosphamide	–	20 (25.3)
Methotrexate	–	26 (32.9)
Mycophenolate Mofetil	–	5 (6.3)
Biologic DMARD treatment	–	14 (17.7)
Adalimumab	–	2 (2.5)
Infliximab	–	9 (11.4)
Rituximab	–	2 (2.5)
Tocilizumab	–	3 (3.8)
Overall audiology outcome	–	
No improvement	–	33 (41.7)
Improvement	–	36 (45.5)
Resolution	–	7 (8.9)
Overall vestibular outcome	–	
No improvement	–	17 (21.5)
Improvement	–	14 (17.7)
Resolution	–	19 (24.0)
Post- treatment right mean PTA (500-4 kHz), in dB	50.7 (37.2)	
Post- treatment left mean PTA (500-4 kHz), in dB	54.6 (38.4)	
Death	–	3 (3.8)

### Steroid resistant vs steroid responsive

Table 2 shows the comparison of characteristics between steroid-resistant (N = 39; 52.0%) and steroid-responsive patients (N = 38; 48.0%). Demographics, clinical characteristics, and lab and imaging findings were similar between the two groups. There was a noticeable difference in the number of patients with dizziness with higher prevalence in the steroid resistant group compared to the steroid responsive group (n = 11; 28.2% vs n = 2; 3.6%). Patients with dizziness have 7.1 times higher odds of being steroid resistant (OR: 7.07, [1.45–34.52, 95% CI]).

**Table 2:** Detailed comparison of demographic, clinical, and audiovestibular profiles in steroid-resistant and steroid-responsive patients

Demographic	Median (25th–75th IQR)	Steroid Resistance Total N = 39		Steroid Responsive Total N = 38	*p* value
		N (%)	Median (25th–75th IQR)	N (%)	
Age (years)	33 (27.0–48.0)	–	36 (25.0–54.0)	–	0.51
Sex					
Male	–	19 (48.7)	–	19 (50.0)	0.91
Female	–	20 (51.3)	–	19 (50.0)	
Race			–		
Caucasian	–	4 (10.2)	–	6 (15.7)	0.69
African American/Black	–	0	–	2 (5.2)	
Asian	–	3 (7.7)	–	4 (10.5)	
Not reported	–	32 (82.0)	–	26 (68.4)	
*Clinical Characteristic*					
Duration of audiovestibular symptom before admissions (months)	1.0 (0.41–3.0)	–	2 (1–10.5)	–	0.12
Presence of systemic symptoms	–	17 (43.5)	–	16 (46.6)	0.89
*Eye symptoms*	–				
Keratitis	–	16 (41.0)	–	12 (31.5)	0.72
Episcleritis/scleritis	–	5 (12.8)	–	8 (21.1)	
Uveitis	–	3 (7.7)	–	3 (7.9)	
Other	–	11 (28.2)	–	12 (31.5)	
Tinnitus	–	20 (51.2)	–	24 (63.2)	0.43
*Subjective hearing loss*					
None	–	3 (7.7)	–	1 (2.6)	0.35
Unilateral	–	7 (17.9)	–	4 (10.5)	
Bilateral	–	29 (74.3)	–	33 (86.8)	
*Vestibular Symptoms*					
Imbalance	–	9 (23.1)	–	14 (36.8)	.03*
Dizziness	–	11 (28.2)	–	2 (5.3)	
Vertigo	–	18 (46.1)	–	18 (47.4)	

Laboratory findings	Mean (SD)	N (%)	Mean (SD)	N (%)	
Erythrocyte sedimentation rate (ESR)	65.2 (35.8)	–	69.9 (33.7)	–	0.55
C-reactive protein (mg/dL)	6.9 (7.1)	–	7.7 (7.0)	–	0.62
Leukocyte count (/mm^3^)	13.4 k (4.4 k)	–	12.9 k (2.9 k)	–	0.56
Hemoglobin (g/dL)	11.3 (2.1)	–	9.8 (2.7)	–	0.03
Platelets (/mm^3^)	327.0 k (283.7 k)	–	443.0 k (154.5 k)	–	0.06
Positive antibody test	–	11 (56.0)	–	11 (28.9)	0.87
Abnormal head imaging	–	9 (37.5)	–	5 (13.2)	0.30

Audiovestibular Test	Mean (SD)	N (%)	Mean (SD)	N (%)	
*Abnormal Caloric Test*					
Diminished	–	2/8(25.0)	–	5/15 (33.3)	0.60
Absent	–	6/8 (75.0)	–	9/15 (60.0)	
Nystagmus	–		–		
Spontaneous	–	1/8 (12.5)	–	4/15 (26.7)	0.38
Positional	–	0/8 (0)	–	1/15 (6.7)	
Not Reported	–	7/8 (87.5)	–	10/15 (66.7)	
Abnormal brainstem auditory evoked response	–	2/8 (25.0)	–	2/15 (13.3)	0.48
Absent stapedial reflex	–	1/8 (12.5)	–	3/15 (20.0)	0.65
Sensorineural hearing loss	–		–		
Unilateral	–	4/27 (14.8)	–	4/31 (12.9)	0.83
Bilateral	–	23/27 (85.1)	–	27/31 (87.1)	
Right mean PTA (500-4 kHz), in dB	76.0 (28.4)	–	59.7 (23.7)	–	0.01*
Left mean PTA (500-4 kHz), in dB	76.0 (27.4)	–	60.1 (25.5)	–	0.01*

We conducted a separate analysis where we combined patients with vertigo, imbalance, or dizziness into one group called vestibular symptoms. Our findings still indicate a notable difference between steroid resistant and steroid responsive patients (n = 31, 79.5% vs. n = 22, 57.9%, p = 0.04). The effect size for this difference was calculated using Cohen’s h, yielding a moderate effect size (h = 0.47). This indicates that the presence of vestibular symptoms has a meaningful impact on the likelihood of being steroid-resistant, beyond just statistical significance. The presence of vestibular symptoms increases the odds of steroid resistance by 181% (OR: 2.81, [1.02–7.73, 95% CI]). In addition, there was a higher right and left mean PTA in the steroid resistant group compared to the steroid responsive group.

### Treatment outcome by treatment group

Table 3 shows the audiological and vestibular outcomes analysis based on treatment (steroids only, conventional DMARD, or biologic DMARD). All patients who received conventional or biologic DMARDs also previously received steroids. More than half of steroid responsive (n = 25; 65.7%) and steroid resistant (n = 23; 60.5%) patients received DMARDs. Higher rates of improvement (64.3%, [36–88%, 95% CI]) and complete resolution (21.4%, [4.8–52%, 95% CI]) in audiological outcomes were seen in the ‘biologic DMARDs’ group. For vestibular outcomes, rates of improvement were similar in both the ‘conventional DMARDs’ group (20.0%, [11.5–33.5%, 95% CI]) and ‘steroid only group (16.6%, [7.3–30.6%, 95% CI]). Higher rate of complete resolution in vestibular outcomes was found in steroid only (30.0%, [14.4–45.0%, 95% CI]). There were no differences in rates of cochlear implants or death and no significant differences in mean PTA results across the different treatment groups. However, both conventional and biologic DMARD patients demonstrated improvement in hearing of about 15 dB, which is the threshold to be considered clinically meaningful, whereas steroid-only patients does not demonstrate this improvement. DMARDs were not started before a median of 1.3 months, whereas the median time to start steroids was 2 weeks (0.5 months).

**Table 3:** Comparative analysis of treatment outcomes with steroids, conventional DMARDs, and biologic DMARDs: audiology, vestibular responses, and additional clinical parameters

Treatment/Outcome	Steroids only N = 30	Conventional DMARDs N = 45	Biologic DMARDs N = 14	p-value
	N (%)	N (%)	N (%)	
*Overall audiology outcome*				
No improvement	18 (60.0)	16 (35.5)	2 (14.2)	0.01*
Improvement	8 (26.7)	26 (57.7)	9 (64.3)	0.01*
Resolution	2 (6.7)	3 (6.6)	3 (21.4)	0.21
Not Reported	2 (6.7)	0	0	–
*Overall vestibular outcome*				
No improvement	7 (23.3)	10 (22.2)	1 (7.1)	0.41
Improvement	5 (16.6)	9 (20.0)	2 (14.2)	0.86
Resolution	9 (30.0)	10 (22.2)	3 (21.4)	0.71
Not Reported	9 (30.0)	16 (35.5)	8 (57.1)	–
Cochlear implants	1 (3.3)	0	0	–
Death	2 (6.7)	1 (2.2)	0	–

	Mean (SD)	Mean (SD)	Mean (SD)	
Change in right mean PTA (500-4 kHz), Mean (SD) in dB	2.43 (34.74)	– 18.9 (19.8)	– 17.6 (15.3)	0.02*
Change in left mean PTA (500-4 kHz), Mean (SD) in dB	4.63 (43.7)	– 16.6 (16.1)	– 12.3 (1.7)	0.001*
Time to treatment (months), Median (25th-75th IQR)	0.5 (0.3–1.3)	1.3 (0.3–3)	1.1 (0.3–12.0)	0.001*

### Treatment characteristics

Table 4 shows further descriptive analysis of audiological and vestibular outcomes by specific conventional and biologic DMARDs. Of the 30 steroid-only patients, 10 (30%) patients reported an auditory improvement of more than 15 dB, but the overall mean from the steroid-only group was below 8 dB. Pooled auditory improvement results showed that treatment with conventional DMARDs had an equivalent rate of auditory improvement (n = 28; 62.2%). Interestingly, cyclophosphamide had the highest auditory improvement rate (n = 12/20; 60%) with a mean (SD) improvement of -16.2 (26.8) dB. In contrast, biologic DMARDs showed a significantly higher rate of auditory improvement (n = 12; 85.7%). When data was separated by steroid resistance, there was a noticeably higher rate of improvement for the steroid responsive group compared to the steroid resistant group (Supplemental Table 4). The pooled results for conventional DMARDs show that 62.1% (n = 18/29) of patients had auditory improvement with a mean (SD) of  – 25.7 (11.1) dB in the steroid responsive group compared to just 45% (n = 9/20) of patients had auditory improvement in the steroid resistant group with a mean (SD) of  – 5.0 (14.9) dB (p = 0.07). For biologic DMARDs, 100% (n = 5/5) of the steroid responsive group had auditory improvement.

**Table 4:** Effectiveness of steroid and DMARD therapies in auditory improvement and resolution: dose overview and response rates

	N	Median (min–max) dose	Auditory improvement* N (%)	Complete auditory resolution N (%)
Steroids	30	–	10/30 (33.3%)	2/30 (6.7%)
*Conventional DMARD*				
Azathioprine	11	150 mg/day (50–160)	3/11 (27.3%)	0
Cyclosporine	6	200 mg/day (120–280)	2/6 (33.3%)	0
Cyclophosphamide	20	1 g/month (0.4–4.0)	12/20 (60%)	2/20 (10.0%)
Methotrexate	26	15 mg/week (7.5–20)	7/26 (26.9%)	1/26 (3.8%)
Mycophenolate Mofetil	5	2 g/day (1–2)	3/4 (75.0%)	1/4 (25.0%)
*Biologic DMARD*				
Adalimumab	2	30 mg/week (20–40)	0	0
Infliximab	9	120 mg/week (50–400)	8/9 (88.9%)	2/9 (22.2%)
Rituximab	2	437.5 mg/week (375–500)	1/2 (50.0%)	0
Tocilizumab	3	162 mg/week (162–640)	3/3 (100%)	2/3 (66.7%)

*If the patient had complete auditory resolution it will also be counted in the N of a auditory improvement

In Supplemental Table 5, mean change after treatment of PTA was reported by treatment group and steroid responsiveness. Steroid responsive patient had a mean (SD) improvement in PTA of  – 17.2 (24.9) when compared to steroid resistant patient who only had mean change in PTA of 2.4 (27.2) across all treatment groups.

Lastly, Supplemental Table 6 shows the response of different ocular manifestations to treatment. Seventeen (60.7%) patients with keratitis, seven (53.8%) patients with scleritis, two (33.3%) patients with uveitis, and eighteen (100%) patients with all other eye symptoms responded to eye and/or systemic treatment. In terms of the overlapping response of the otologic symptoms, we found that 16 (57.1%) patients with keratitis, 9 (69.2%) patients with scleritis, 3 (50.0%) patients with uveitis, and 11 (61.1%) patients with all other eye symptoms had audiological improvements.

## Discussion

Cogan syndrome is a rare chronic systemic inflammatory disorder that affects the vestibular and hearing system. There are no clear guidelines or management of CS because of its rarity and limited research. This is also to be expected as a power analysis conducted in 2006 by Wei et al. showed that a study would need 1,000 patients to be able to detect an improvement in the recovery rate of more than 10% over spontaneous recovery [16]. Multiple factors limit the viability of such research endeavors including small sample size, multiple etiologies of hearing loss and vertigo, time to treatment, etc. As a result, it is challenging to establish evidence-based guidelines for treating audiovestibular manifestations of rare disorders like CS. The purpose of this review is to better characterize resistance to steroids and the use of DMARDs in patients with Cogan syndrome.

### Rationale for corticosteroids and DMARDs

Steroids are commonly used in Cogan syndrome, traditionally thought to address autoimmune-related inflammation in the inner ear. However, multiple studies have shown that this is only sometimes the case [17–21]. It is believed that the systemic inflammatory response can lead to disruption of the vascular endothelial integrity, which causes a breakdown in the blood labyrinth barrier and, subsequently, endolymph homeostasis [22–24]. This can affect the endocochlear potential and hearing. Steroids may temporarily help by regulating tight junctions impacting this potential [25]. Biologic DMARDs, as second-line treatments, could reduce the inflammation but these are expensive drugs that have significant side effects, including increasing the risk of infections and malignancies. There’s limited evidence of their effectiveness in autoimmune inner ear diseases, and more research is needed; however, this systematic review suggests that they can be effective [26–28]. Multidisciplinary collaboration is needed with rheumatologists, ophthalmologists, and neurotologists to be able to manage these patients, both responsive and resistant to steroid treatment.

### Assessment and implications of treatment strategies: steroid resistance, DMARD efficacy, and vestibular symptoms

Currently, the first line treatment for Cogan syndrome is steroids, but no double-blinded controlled studies have indicated the benefits of its use [2, 9, 29]. Steroids are known to be quite effective for many disease processes in the short term. However, they are not long-term solutions as they can have serious long-term side effects such as Cushingoid syndrome, vascular complications, and metabolic derangements [7]. Furthermore, the literature has also pointed out instances of CS patients failing steroid treatment, defined as two weeks of no response to steroid administration [6, 8, 9]. When this happens, DMARDs are often second-line therapies, but they are not initiated for weeks as clinicians wait to see if patients first respond to steroids. However, if clinicians knew which patients were at high risk for steroid therapy failure early on, they could initiate second-line therapies sooner for quicker symptomatic remission.

Based on our results, patients with dizziness had higher odds to be steroid resistant. In the compiled case reports, “dizziness” referred to as a complaint that did not specifically cite vertigo or imbalance. Because of this lack of further characterization, we grouped these similar symptoms into a single category called vestibular symptoms. There was still a noticeable difference in the rates of these vestibular symptoms between the steroid resistant and steroid responsive groups (n = 31, 79.5% vs n = 22, 57.9%). With the presence of vestibular symptoms increasing the odds of steroid resistance by 181%. This association might be because these symptoms often indicate more severe involvement of the inner ear. The inner ear’s complex anatomy, along with limited blood supply, might reduce drug delivery and effectiveness. Furthermore, severe inflammation in the inner ear with potential scarring can cause lasting damage and decrease corticosteroid treatment efficacy. This suggests that vestibular symptoms, such as dizziness, vertigo, or imbalance, could be useful clinical characteristics when identifying patients at risk for steroid resistance who would probably benefit from earlier initiation of DMARD therapy. Future studies should focus on characterizing patients’ vestibular symptoms to see if there might be any associations with steroid responsiveness for CS patients. At this time, no apparent risk factor exists that can allow us to predict who will be responsive to steroids beforehand.

Regarding treatment, both steroid-resistant and steroid-responsive CS patients were analyzed. Our findings were consistent with a 2015 study by Brant et al. in terms of responses to cyclophosphamide, methotrexate, and infliximab [30]. However, our study showed a lower response rate to azathioprine compared to Brant et al. Importantly, Brant et al.’s research focused on autoimmune inner ear disease, not exclusively on CS. Our data aligns with previous literature, suggesting a superior response to biologic DMARDs in CS [11, 31]. Our findings suggest biologic DMARDs were associated with higher audiological improvement compared to steroids alone. This could be due to the targeted and sustained action of biologic DMARDs in reducing inner ear inflammation and preventing immune-mediated damage. Patients on biologic DMARDs showed significant improvement in hearing, a pattern not observed in the steroids-only group. However, more data is needed to conclude if any treatment is superior for vestibular recovery. Steroid resistant patients showed a lower rate of auditory improvement with DMARD treatment than steroid responsive patients. Despite late DMARD administration, steroid-responsive patients still had better auditory outcomes, suggesting inherent aspects of steroid resistance may lead to poorer auditory results. Additionally, only a few studies have reported data on the time from the onset of vestibular symptoms to the initiation of treatment. In our study, the median time to treatment for DMARDs is more than doubled that of steroids only (1.3 months vs 0.5 months).

However, the optimal timing of DMARD initiation remains to be determined. Is important to note that all patients who received conventional or biologic DMARDs also previously received steroids. Steroids could potentially have acted as priming agents for DMARDs by initially reducing inflammation and allowing DMARDs to be more effective in maintaining and furthering the anti-inflammatory effect. Future investigations should focus on how the timing of DMARD initiation affects patient outcomes.

Regarding ocular symptoms, a better response rate was seen when compared to audiological and vestibular symptoms. A previous study has found that ocular symptoms in Cogan Syndrome tend to recover more readily than audiovestibular symptoms because ocular inflammation responds well to corticosteroids and immunosuppressants, whereas the complex nature of inner ear inflammation makes audiovestibular symptoms harder to treat effectively [32]. Additionally, it is important to note the difference between the blood-retinal and blood-labyrinthine barriers. According to Ishiyama et al., both barriers can break down due to inflammation, but the permeability of the cochlear and vestibular barriers may be less than that of the retinal barrier [33]. This reduced permeability could mean that medications are less likely to diffuse into the inner ear, allowing more time for scarring to set in, which could contribute to the differential response to treatment. [33]

### Proposed treatment algorithm

In response to the limited efficacy of steroids and conventional DMARDs in some patients with Cogan syndrome, we propose a preliminary decision-making algorithm. Initially, patients diagnosed with Cogan syndrome should be assessed for their response to high-dose corticosteroids (0.5–2.0 mg/kg/day) administered within the first two weeks. If there is an improvement of at least 15 dB in hearing, corticosteroid treatment should be continued with adjustments based on ongoing symptom evaluation and continued treatment and tapering over a period of at least 2 months. For patients showing no improvement or relapse during tapering, the next step would be to integrate conventional DMARDs, such as methotrexate or azathioprine, monitoring the response over a period of one to three months. If patients remain non-responsive or show inadequate response to these treatments, the introduction of biologic DMARDs, such as infliximab or adalimumab, should be considered. Given the sparse data on biologic DMARDs, their use should be contemplated on a case-by-case basis, preferably under rigorous clinical supervision. Throughout the treatment process, continuous monitoring of auditory and vestibular symptoms is crucial, with adjustments made as needed to optimize therapeutic outcomes. Moreover, all treatment outcomes and adjustments should be meticulously documented to aid in accumulating a robust database for future research on Cogan syndrome. This algorithm is based on limited evidence and a small sample size because of that we recommend that clinicians apply this algorithm with discretion, tailored to individual patient circumstances and in conjunction with clinical judgment.

### Limitations

All included studies in this review were case reports and case series. Thus, our study was limited by the sample size and heterogeneity of the included studies as well as by the fact that the study design lacked high-quality evidence. Consequently, the results of this review should be interpreted with increased caution. Certain studies also did not further characterize the etiologies behind vestibular symptoms, such as vertigo, imbalance, hemodynamic causes, etc., which further limited the results of this study. A limitation of our study is the inclusion of case reports and series that did not consistently exclude other comorbid conditions. This lack of baseline information could influence the interpretation of steroid responsiveness. Pre-existing conditions, such as previous sensorineural hearing loss or vestibular disorders, could predispose patients to poorer outcomes and reduced responsiveness to steroids. This potential confounder must be mentioned as it may partly explain why some patients did not respond to treatment as expected. Another limitation of our review is the insufficient initial phenotype data, limiting our ability to detail Cogan syndrome’s varied presentations which is important for earlier diagnosis. In addition, vestibular function tests in the review only included caloric testing due to lack of others. It is now well-recognized that vestibular weakness can be frequency selective. Therefore, relying solely on caloric testing, which assesses only a small fraction of the vestibular system, needs to comprehensively assess vestibular weakness. Additionally, some studies did not perform repeat vestibular function tests after treatment, relying primarily on symptom relief as an outcome measure. This reliance on subjective symptom relief is insufficient to infer complete recovery of the underlying pathology, as it does not provide objective evidence of improvement in vestibular function. However, with the advent of new techniques to gauge vestibular function, such as video head impulse testing, future studies should take into account vestibular testing as objective outcome measures that can be obtained at pre and post-treatment. Despite these limitations, this review still provides crucial information and contributes to the limited literature surrounding Cogan syndrome. Additionally, the retrospective nature of this study only permits associations to be drawn rather than causations. Future prospective studies are needed to better distinguish patients with steroid resistance from those with steroid responsiveness and to evaluate the optimal timing for the initiation of DMARDs. However, given the rarity of Cogan syndrome, conducting large-scale prospective studies may be challenging. Therefore, collaborative multicenter studies, patient registries, and the use of advanced statistical methods to analyze existing case series and retrospective data such as this study could provide valuable insights into the management of this rare condition.

## Conclusion

Our study synthesized the available literature to better characterize patients with steroid resistance and those without steroid resistance. Vestibular symptoms were noted to be more prevalent in patients who were eventually labeled as steroid resistant. Furthermore, we found higher rates of audiological improvement in patients given biologic DMARDs rather than conventional DMARD or steroids only. However, further studies are needed to characterize and better define vestibular symptoms and investigate the utility and timing of biologic DMARDs in managing patients with Cogan syndrome.

## Supplementary Information

Below is the link to the electronic supplementary material.

Supplementary file1 (DOCX 153 KB)

Supplementary file2 (DOCX 25 KB)

## Data Availability

The datasets generated and/or analyzed during the current study are not publicly available but are available from the corresponding author on reasonable request.
