# Supplementary material for: Audiovestibular outcomes in adult patients with cogan syndrome: a systematic review
Source: Eur Arch Otorhinolaryngol. 2024 Aug 7;282(1):23–35. doi: 10.1007/s00405-024-08878-5 (PMC11735566; doi:10.1007/s00405-024-08878-5)
Supplement: Supplementary file 1 — Supplementary file1 (DOCX 153 KB) [file 405_2024_8878_MOESM1_ESM.docx]

**Supplemental Table 1**. PICOTS table

| PICOTS Element | Description |
| --- | --- |
| Population | Adults patients diagnosed with Cogan syndrome |
| Intervention | Use of steroid only |
| Comparison | Use of DMARDs (Disease-Modifying Anti-Rheumatic Drugs) |
| Outcome | - Audiological and vestibular data  - Type of pharmacologic treatment given |
|  | - Rates of symptoms improvements or resolution |
|  | - Other lab values |
| Timing | - Time to treatment initiation |
|  | - Within 2 weeks of steroid administration for responsiveness |
| Setting | Data collected from case reports and case series |

**Supplemental Table 2**. Critical Appraisal of Case Reports

| Author | Year | Quality^a^ | JBI 7.4.1^b^ | 7.4.2^c^ | 7.4.3^d^ | 7.4.4^e^ | 7.4.5^f^ | 7.4.6^g^ | 7.4.7^h^ | 7.4.8^i^ | Total Score |
| --- | --- | --- | --- | --- | --- | --- | --- | --- | --- | --- | --- |
| Albright et al.^1^ | 1961 | Y | Y | Y | Y | Y | N | Y | Y | N | 7 |
| Azami et al.^2^ | 2014 | Y | Y | N | Y | Y | Y | N | N | Y | 6 |
| Bauman et al.^3^ | 2005 | Y | Y | Y | Y | Y | Y | Y | Y | Y | 9 |
| Bauman et al.^3^ | 2005 | Y | Y | Y | Y | N | Y | Y | Y | Y | 8 |
| Bauman et al.^3^ | 2005 | Y | Y | Y | Y | Y | Y | Y | Y | Y | 9 |
| Beccastrini et al. ^4^ | 2005 | Y | Y | N | N | N | Y | N | N | Y | 4 |
| Beccastrini et al. ^4^ | 2005 | Y | Y | N | N | N | Y | N | N | Y | 4 |
| Beccastrini et al. ^4^ | 2005 | Y | Y | N | N | N | Y | N | N | Y | 4 |
| Belluci et al.^5^ | 1974 | Y | Y | Y | Y | Y | Y | Y | Y | Y | 9 |
| Benitez et al.^6^ | 1990 | Y | Y | Y | Y | Y | Y | Y | N | Y | 8 |
| Best et al.^7^ | 2013 | Y | Y | Y | Y | Y | N | N | Y | Y | 7 |
| Bhandari et al.^8^ | 2019 | Y | Y | Y | Y | Y | N | Y | Y | Y | 8 |
| Boyd et al.^9^ | 1957 | Y | Y | Y | Y | Y | N | N | Y | Y | 7 |
| Boyd et al.^9^ | 1957 | Y | Y | Y | Y | Y | N | N | Y | Y | 7 |
| Bunker et al.^10^ | 2016 | Y | Y | Y | Y | Y | Y | Y | Y | Y | 9 |
| Cabezas-Rodriguez et al.^11^ | 2017 | Y | Y | Y | Y | Y | Y | Y | Y | Y | 9 |
| Cassis et al.^12^ | 2018 | Y | Y | Y | Y | N | N | Y | Y | Y | 7 |
| Cochrane et al.^13^ | 1991 | Y | Y | Y | Y | Y | N | N | Y | Y | 7 |
| Cote et al.^14^ | 1993 | Y | Y | Y | Y | Y | Y | Y | Y | Y | 9 |
| Cundiff et al.^15^ | 2006 | Y | Y | Y | Y | Y | Y | Y | Y | Y | 9 |
| Dekker et al.^16^ | 1996 | Y | Y | Y | Y | N | N | Y | Y | Y | 7 |
| DelCarpio et al.^17^ | 1976 | Y | Y | Y | Y | N | Y | N | Y | Y | 7 |
| Djupesland et al.^18^ | 1974 | Y | Y | Y | Y | Y | Y | Y | Y | Y | 9 |
| Djupesland et al.^18^ | 1974 | Y | Y | Y | Y | Y | Y | Y | Y | Y | 9 |
| Edrees et al.^19^ | 2003 | Y | Y | Y | Y | N | N | Y | Y | Y | 7 |
| Fidler et al.^20^ | 1989 | Y | Y | Y | Y | Y | Y | Y | Y | Y | 9 |
| Forli et al.^21^ | 2009 | Y | Y | Y | Y | Y | Y | Y | Y | Y | 9 |
| Fricker et al.^22^ | 2007 | Y | Y | Y | Y | Y | Y | Y | Y | Y | 9 |
| Fricker et al.^22^ | 2007 | Y | Y | Y | Y | Y | Y | Y | Y | Y | 9 |
| Georgakopoulos et al.^23^ | 2014 | Y | Y | Y | Y | Y | Y | Y | Y | Y | 9 |
| Ghadban et al.^24^ | 2008 | Y | Y | Y | Y | Y | Y | Y | Y | Y | 9 |
| Goncalves et al.^25^ | 2004 | Y | Y | Y | Y | Y | Y | Y | Y | Y | 9 |
| Hafner et al.^26^ | 2021 | Y | Y | Y | Y | Y | Y | Y | Y | Y | 9 |
| Hara et al.^27^ | 2021 | Y | Y | Y | Y | Y | Y | Y | Y | Y | 9 |
| Hirvonen et al.^28^ | 2013 | Y | Y | Y | Y | Y | Y | Y | Y | Y | 9 |
| Hurelbrink et al.^29^ | 2011 | Y | Y | Y | Y | N | N | N | N | Y | 5 |
| Ikeda et al.^30^ | 2002 | Y | Y | Y | N | N | Y | N | N | Y | 5 |
| Im et al.^31^ | 2007 | Y | Y | Y | Y | N | N | N | N | Y | 5 |
| Jung et al.^32^ | 2016 | Y | Y | Y | Y | Y | Y | Y | Y | Y | 9 |
| Kamakura et al.^33^ | 2017 | Y | Y | Y | Y | Y | Y | Y | N | Y | 8 |
| Karni et al.^34^ | 1991 | Y | Y | Y | Y | N | N | Y | N | Y | 6 |
| Kawasaki et al.^35^ | 2018 | Y | Y | Y | Y | N | Y | Y | N | Y | 7 |
| Klement et al.^36^ | 2007 | Y | Y | Y | Y | N | N | N | Y | Y | 6 |
| Kondo et al.^37^ | 2009 | Y | Y | Y | Y | Y | Y | Y | N | Y | 8 |
| Kougkas et al.^38^ | 2021 | Y | Y | Y | Y | N | Y | Y | N | Y | 7 |
| Lee et al.^39^ | 2019 | Y | Y | Y | Y | Y | Y | Y | N | Y | 8 |
| Lepur et al.^40^ | 2004 | Y | N | Y | Y | Y | Y | Y | Y | N | 7 |
| Lima et al.^41^ | 2006 | Y | Y | Y | Y | Y | Y | Y | Y | Y | 9 |
| Maalikjy et al.^42^ | 2000 | Y | Y | Y | N | N | Y | N | N | Y | 5 |
| Maikap et al.^43^ | 2021 | Y | Y | Y | Y | Y | Y | Y | Y | Y | 9 |
| Manto et al.^44^ | 1996 | Y | Y | Y | Y | Y | Y | N | N | Y | 7 |
| Migliori et al.^45^ | 2009 | Y | Y | Y | Y | Y | Y | Y | Y | Y | 9 |
| Miserocchi et al.^46^ | 2001 | Y | Y | Y | N | Y | N | N | N | Y | 5 |
| Montes et al.^47^ | 2014 | Y | Y | Y | Y | Y | Y | Y | N | Y | 8 |
| Morinaka et al.^48^ | 2020 | Y | Y | Y | Y | Y | Y | N | N | Y | 7 |
| Ndiaye et al.^49^ | 2002 | Y | Y | Y | Y | Y | Y | Y | N | Y | 8 |
| Orsoni et al.^50^ | 2010 | Y | Y | Y | Y | Y | Y | Y | N | Y | 8 |
| Peeters et al.^51^ | 1986 | Y | Y | Y | Y | Y | Y | Y | N | Y | 8 |
| Phee et al.^52^ | 2017 | Y | Y | Y | Y | N | N | N | N | Y | 5 |
| Pherwani et al.^53^ | 2008 | Y | Y | Y | Y | Y | Y | Y | N | Y | 8 |
| Pouchot et al.^54^ | 1995 | Y | Y | Y | Y | Y | Y | N | Y | Y | 8 |
| Queiros et al.^55^ | 2013 | Y | Y | Y | Y | Y | Y | Y | Y | Y | 9 |
| Raza et al.^56^ | 1998 | Y | Y | Y | Y | Y | Y | Y | Y | Y | 9 |
| Richardson et al.^57^ | 1994 | Y | Y | Y | Y | Y | Y | Y | Y | Y | 9 |
| Shibuya et al.^58^ | 2013 | Y | Y | Y | Y | Y | Y | Y | Y | Y | 9 |
| Takashi et al.^59^ | 2018 | Y | Y | Y | Y | Y | N | Y | Y | Y | 8 |
| Teece et al.^60^ | 2011 | Y | Y | Y | Y | Y | N | Y | N | Y | 7 |
| Teece et al.^60^ | 2011 | Y | Y | Y | Y | Y | N | Y | N | Y | 7 |
| Togashi et al.^61^ | 2008 | Y | Y | Y | Y | Y | Y | Y | Y | Y | 9 |
| Touma et al.^62^ | 2007 | Y | Y | Y | Y | Y | Y | Y | Y | Y | 9 |
| Touma et al.^62^ | 2007 | Y | Y | N | Y | Y | Y | Y | Y | Y | 8 |
| Trevino Gonzalez et al.^63^ | 2018 | Y | Y | Y | Y | Y | Y | Y | Y | Y | 9 |
| Van Doornum et al.^64^ | 2001 | Y | Y | Y | Y | Y | Y | Y | N | Y | 8 |
| Van Doornum et al.^64^ | 2001 | Y | Y | Y | Y | Y | Y | N | N | Y | 7 |
| Vasileiadis et al.^65^ | 2012 | Y | Y | Y | Y | Y | Y | Y | N | Y | 8 |
| Vishwakarma et al.^66^ | 2007 | Y | Y | Y | Y | N | Y | Y | Y | Y | 8 |
| Watanabe et al.^67^ | 2000 | Y | Y | Y | Y | Y | Y | Y | N | Y | 8 |
| Wilder-Smith et al.^68^ | 1990 | Y | Y | Y | Y | Y | Y | N | Y | Y | 8 |
| Yaginuma et al.^69^ | 2009 | Y | Y | Y | Y | Y | Y | N | Y | Y | 8 |
| Ying et al.^70^ | 2010 | Y | Y | Y | Y | Y | Y | Y | Y | Y | 9 |

^a^per Oxford Center for Evidence-Based Medicine criteria; ^b^Joanna Briggs Institute Manual for Evidence Synthesis risk of bias question 7.4.1: Were patient’s demographic characteristics clearly described?; ^c^Was the patients history clearly described and presented as a timeline?; ^d^Was the current clinical condition of the patient on presentation clearly described?; ^e^Were diagnostic tests or assessment methods and the results clearly described?; ^f^Was the intervention(s) or treatment procedure(s) clearly described?; ^g^Was the post-intervention clinical condition clearly described?; ^h^Were adverse events (harms) or unanticipated events identified and described?; ^i^Does the case report provide takeaway lessons?

**Supplemental Table 3**. Summary of Included Studies

| Author | Year | Age^a^, Sex^b^ | Steroid resistant? | Systemic symptoms? | Treatment | Auditory Outcome | Vestibular Outcome |
| --- | --- | --- | --- | --- | --- | --- | --- |
| Albright et al.^1^ | 1961 | 25, M | No | No | C-DMARD | No improvement | No improvement |
| Azami et al.^2^ | 2014 | 47, F | - | Yes | C-DMARD | Improvement | Improvement |
| Bauman et al.^3^ | 2005 | 33, M | Yes | No | C-DMARD | Improvement | Resolution |
| Bauman et al.^3^ | 2005 | 48, F | Yes | Yes | C-DMARD | No improvement | No improvement |
| Bauman et al.^3^ | 2005 | 49, M | No | Yes | C-DMARD | Improvement | - |
| Beccastrini et al. ^4^ | 2005 | 30, F | Yes | No | C-DMARD, B-DMARD | Improvement | - |
| Beccastrini et al. ^4^ | 2005 | 29, M | Yes | No | B-DMARD | Resolution | - |
| Beccastrini et al. ^4^ | 2005 | 35, M | Yes | No | C-DMARD, B-DMARD | Improvement | Improvement |
| Belluci et al.^5^ | 1974 | 21, F | Yes | No | Steroids Only | No improvement | Improvement |
| Benitez et al.^6^ | 1990 | 30, F | No | No | Steroids Only | Resolution | Resolution |
| Best et al.^7^ | 2013 | 31, F | Yes | No | C-DMARD | No improvement | No improvement |
| Bhandari et al.^8^ | 2019 | 21, M | Yes | Yes | C-DMARD | No improvement | Resolution |
| Boyd et al.^9^ | 1957 | 28, M | Yes | Yes | C-DMARD | Improvement | Improvement |
| Boyd et al.^9^ | 1957 | 27, M | Yes | Yes | Steroids Only | Improvement | Improvement |
| Bunker et al.^10^ | 2016 | 43, F | Yes | Yes | C-DMARD, B-DMARD | No improvement | Resolution |
| Cabezas-Rodriguez et al.^11^ | 2017 | 82, F | Yes | Yes | C-DMARD | No improvement | Resolution |
| Cassis et al.^12^ | 2018 | 24, F | Yes | No | C-DMARD | No improvement | Resolution |
| Cochrane et al.^13^ | 1991 | 35, M | Yes | Yes | Steroids Only | No improvement | No improvement |
| Cote et al.^14^ | 1993 | 41, F | Yes | Yes | Steroids Only | No improvement | - |
| Cundiff et al.^15^ | 2006 | 25, M | No | No | Steroids Only | No improvement | - |
| Dekker et al.^16^ | 1996 | 51, F | Yes | Yes | Steroids Only | No improvement | - |
| DelCarpio et al.^17^ | 1976 | 58, M | Yes | Yes | Steroids Only | No improvement | - |
| Djupesland et al.^18^ | 1974 | 19, F | No | No | Steroids Only | No improvement | Resolution |
| Djupesland et al.^18^ | 1974 | 25, M | No | No | Steroids Only | Improvement | Resolution |
| Edrees et al.^19^ | 2003 | 37, M | No | Yes | C-DMARD | Improvement |  |
| Fidler et al.^20^ | 1989 | 62, F | No | No | Steroids Only | Improvement | Improvement |
| Forli et al.^21^ | 2009 | 54, F | No | No | Steroids Only | - | - |
| Fricker et al.^22^ | 2007 | 33, M | Yes | No | C-DMARD, B-DMARD | Improvement | Resolution |
| Fricker et al.^22^ | 2007 | 49, M | No | No | B-DMARD | Improvement | - |
| Georgakopoulos et al.^23^ | 2014 | 62, F | No | No | Steroids Only | Improvement | - |
| Ghadban et al.^24^ | 2008 | 48, F | No | Yes | C-DMARD, B-DMARD | Resolution | - |
| Goncalves et al.^25^ | 2004 | 53, M | No | Yes | Steroids Only | Improvement | Resolution |
| Hafner et al.^26^ | 2021 | 41, F | No | Yes | C-DMARD, B-DMARD | Improvement | - |
| Hara et al.^27^ | 2021 | 49, M | Yes | Yes | B-DMARD | Resolution | - |
| Hirvonen et al.^28^ | 2013 | 28, F | No | No | C-DMARD | Improvement | Resolution |
| Hurelbrink et al.^29^ | 2011 | 33, M | Yes | Yes | C-DMARD | No improvement | No improvement |
| Ikeda et al.^30^ | 2002 | 61, M | Yes | Yes | Steroids Only | No improvement | No improvement |
| Im et al.^31^ | 2007 | 25, F | Yes | No | C-DMARD | No improvement | No improvement |
| Jung et al.^32^ | 2016 | 76, F | - | Yes | Steroids Only | - | - |
| Kamakura et al.^33^ | 2017 | 64, M | Yes | No | Steroids Only | No improvement | Improvement |
| Karni et al.^34^ | 1991 | 32, F | Yes | No | Steroids Only | No improvement | Improvement |
| Kawasaki et al.^35^ | 2018 | 27, F | No | Yes | Steroids Only | Resolution | Resolution |
| Klement et al.^36^ | 2007 | 18, F | No | No | C-DMARD | No improvement | Improvement |
| Kondo et al.^37^ | 2009 | 59, F | No | Yes | Steroids Only | Improvement | Resolution |
| Kougkas et al.^38^ | 2021 | 25, M | No | No | C-DMARD, B-DMARD | Improvement | Resolution |
| Lee et al.^39^ | 2019 | 60, M | No | Yes | C-DMARD | No improvement | Improvement |
| Lepur et al.^40^ | 2004 | 20, NA | Yes | No | Steroids Only | No improvement | - |
| Lima et al.^41^ | 2006 | 32, F | Yes | Yes | C-DMARD | Improvement | - |
| Maalikjy et al.^42^ | 2000 | 33, F | No | No | C-DMARD | Improvement | Improvement |
| Maikap et al.^43^ | 2021 | 35, M | No | No | C-DMARD | Improvement | - |
| Manto et al.^44^ | 1996 | 22, M | Yes | No | Steroids Only | No improvement | No improvement |
| Migliori et al.^45^ | 2009 | 31, F | Yes | No | C-DMARD | Improvement | No improvement |
| Miserocchi et al.^46^ | 2001 | 28, M | Yes | No | Steroids Only | No improvement | No improvement |
| Montes et al.^47^ | 2014 | 44, M | No | Yes | C-DMARD | Resolution | - |
| Morinaka et al.^48^ | 2020 | 53, F | Yes | Yes | Steroids Only | No improvement | - |
| Ndiaye et al.^49^ | 2002 | 18, M | No | Yes | C-DMARD | Improvement | Improvement |
| Orsoni et al.^50^ | 2010 | 25, F | Yes | No | C-DMARD, B-DMARD | Improvement | - |
| Peeters et al.^51^ | 1986 | 20, F | No | No | C-DMARD | Improvement | No improvement |
| Phee et al.^52^ | 2017 | 32, M | No | Yes | C-DMARD | Improvement | No improvement |
| Pherwani et al.^53^ | 2008 | 54, M | No | No | C-DMARD | Improvement | - |
| Pouchot et al.^54^ | 1995 | 33, F | No | No | C-DMARD | Improvement | Improvement |
| Queiros et al.^55^ | 2013 | 23, M | Yes | Yes | C-DMARD | No improvement | - |
| Raza et al.^56^ | 1998 | 58, F | No | Yes | C-DMARD | Resolution | - |
| Richardson et al.^57^ | 1994 | 29, M | No | No | C-DMARD | Improvement | - |
| Shibuya et al.^58^ | 2013 | 69, M | No | Yes | C-DMARD, B-DMARD | Improvement | - |
| Takashi et al.^59^ | 2018 | 69, F | No | Yes | Steroids Only | No improvement | Resolution |
| Teece et al.^60^ | 2011 | 23, F | No | No | Steroids Only | Improvement | No improvement |
| Teece et al.^60^ | 2011 | 30, M | Yes | No | Steroids Only | No improvement | No improvement |
| Togashi et al.^61^ | 2008 | 63, M | No | No | C-DMARD | Improvement | - |
| Touma et al.^62^ | 2007 | 37, F | Yes | No | B-DMARD | Improvement | Improvement |
| Touma et al.^62^ | 2007 | 34, F | Yes | No | C-DMARD, B-DMARD | No improvement | No improvement |
| Trevino Gonzalez et al.^63^ | 2018 | 41, M | No | No | Steroids Only | Improvement | Resolution |
| Van Doornum et al.^64^ | 2001 | 30, M | No | Yes | C-DMARD | Improvement | Improvement |
| Van Doornum et al.^64^ | 2001 | 36, M | Yes | Yes | Steroids Only | No improvement | No improvement |
| Vishwakarma et al.^66^ | 2007 | 58, F | Yes | No | C-DMARD | No improvement | No improvement |
| Watanabe et al.^67^ | 2000 | 18, F | No | Yes | C-DMARD | Improvement | Resolution |
| Wilder-Smith et al.^68^ | 1990 | 20, F | Yes | No | C-DMARD | No improvement | - |
| Yaginuma et al.^69^ | 2009 | 59, F | Yes | No | Steroids Only | No improvement | Resolution |
| Ying et al.^70^ | 2010 | 51, F | No | No | C-DMARD | No improvement | Resolution |

Supplemental Table 4. Comparison of Auditory Symptom Response and Resolution in Steroid-Resistant and Steroid-Responsive Patients Undergoing Various Treatments

|  | Steroid Resistant | | Steroid Responsive | |
| --- | --- | --- | --- | --- |
|  | Auditory improvement  N (%) | Complete auditory resolution  N (%) | Auditory improvement  N (%) | Complete auditory resolution  N (%) |
| Steroids | 0 | 0 | 7/13 (53.8%) | 2/13 (15.3%) |
| Conventional DMARD |  |  |  |  |
| Azathioprine | 0 | 0 | 3/4 (75%) | 0 |
| Cyclosporine | 0 | 0 | 2/3 (66.6%) | 0 |
| Cyclophosphamide | 4/9 (33.3%) | 0 | 8/9 (88.9%) | 2/9 (22.2%) |
| Methotrexate | 4/10 (20%) | 0 | 3/10 (30%) | 1/14 (7.1%) |
| Mycophenolate Mofetil | 1/1 (100.0%) | 0 | 2/3 (66.7%) | 1/3 (33.3%) |
| Pooled Results | 9/20 (45.0%) | 0 | 18/29 (62.1%) | 3/29 (10.3%) |
| Biologic DMARD |  |  |  |  |
| Adalimumab | 0 | 0 | 0 | 0 |
| Infliximab | 5/6 (83.3%) | 1/6 (16.7%) | 3/3 (100.0%) | 1/3 (33.3%) |
| Rituximab | 1/2 (50.0%) | 0 | - | - |
| Tocilizumab | 1/1 (100.0%) | 1/1 (100.0%) | 2/2 (100.0%) | 1/2 (50.0%) |
| Pooled Results | 7/9 (77.8%) | 2/7 (28.6%) | 5/5 (100.0%) | 2/5 (40.0%) |

Supplemental Table 5: Changes in Mean PTA by Treatment Group and Steroid Responsiveness

|  | Change in mean PTA (500-4kHz), Mean (SD) in dB | | |
| --- | --- | --- | --- |
|  | Overall | Steroid Resistant | Steroid Responsive |
| All Treatment Groups | -9.8 (27.6) | 2.4 (27.2) | -17.2 (24.9)* |
| Steroid Only | 3.6 (38.4) | 32.4 (33.3) | -6.8 (35.8) |
| Conventional DMARD | -17.8 (16.2)* | -5.0 (14.9) | -25.7 (11.5)* |
| Biologic DMARD | -14.8 (10.6) | -12.2 (10.5) | -21.3 (11.8)* |
| *Denotes clinically significant improvement of more than 15dB | | | |

Supplemental Table 6: Response Rates to Treatment in Patients with Eye Symptoms and Associated Auditory Improvement

|  | Total  N | Response to eye and/or systemic treatment  N (%) | Total  N | Auditory improvement  N (%) |
| --- | --- | --- | --- | --- |
| Eye Symptoms |  |  |  |  |
| Keratitis | 28 | 17 (60.7%) | 28 | 16 (57.1%) |
| Episcleritis/scleritis | 13 | 7 (53.8%) | 13 | 9 (69.2%) |
| Uveitis | 6 | 2 (33.3%) | 6 | 3 (50.0%) |
| All other eye symptoms | 18 | 18 (100.0%) | 18 | 11 (61.1%) |
