# Supplementary material for: Audiovestibular outcomes in adult patients with cogan syndrome: a systematic review
Source: Eur Arch Otorhinolaryngol. 2024 Aug 7;282(1):23–35. doi: 10.1007/s00405-024-08878-5 (PMC11735566; doi:10.1007/s00405-024-08878-5)
Supplement: Supplementary file 2 — Supplementary file2 (DOCX 25 KB) [file 405_2024_8878_MOESM2_ESM.docx]

Appendix A – Search Terms

| Database | Search Terms |
| --- | --- |
| Pubmed: 08/01/2023 (477 results) | ("Cogan syndrome"[Mesh] OR cogan OR cogan’s) AND (audiovestibular OR audio* OR dizziness OR vertigo OR vestibular OR "bilateral vestib*" OR "Endolymphatic Hydrops"[Mesh] OR "Delayed endolymphatic hydrops" OR "Hearing Loss"[Mesh] OR "Audiometry"[Mesh] OR "Vestibular Diseases"[Mesh] OR "Vestibular System"[Mesh] OR "Vestibular Evoked Myogenic Potentials"[Mesh] OR "Vestibular Function Tests"[Mesh] OR "Acoustic Impedance Tests"[Mesh] OR "Evoked Potentials, Auditory, Brain Stem"[Mesh] OR "Otoacoustic Emissions, Spontaneous"[Mesh] OR "tympanom*" OR hearing OR "vestibu*" OR vertigo OR "dizz*" OR "calori*" OR "tinnitus" OR "audiom*" OR "pure-tone" OR "otoacoustic emissions" OR "DPOAE" OR "Auditory brainstem response") |
| SCOPUS – 581 (08/01/2023) | TITLE-ABS-KEY ( {Cogan syndrome}  OR  cogan )  AND  TITLE-ABS-KEY ( audiovestibular  OR  audio*  OR  dizziness  OR  vertigo  OR  vestibular  OR  {bilateral vestib*}  OR  {Endolymphatic Hydrops}  OR  {Delayed endolymphatic hydrops}  OR  {Hearing Loss}  OR  audiometry  OR  {Vestibular Diseases}  OR  {Vestibular Evoked Myogenic Potentials}  OR  {Vestibular Function Tests}  OR  {Acoustic Impedance Tests}  OR  {Evoked Potentials}  OR  {Auditory Brain Stem}  OR  {Otoacoustic Emissions}  OR  tympanom*  OR  hearing  OR  vestibu*  OR  vertigo  OR  dizz*  OR  calori*  OR  tinnitus  OR  audiom*  OR  {pure-tone}  OR  {pure tone}  OR  {otoacoustic emissions}  OR  dpoae  OR  {Auditory brainstem response} ) |
| CINAHL – 70 results (08/01/23) | S1: audiovestibular OR audio OR dizziness OR vertigo OR vestibular OR bilateral vestibular OR endolymphatic hydrops OR delayed endolymphatic hydrops OR hearing loss OR audiometry OR vestibular diseases OR vestibular evoked myogenic potential  S2: (vestibular function tests OR acoustic impedance OR evoked potentials OR auditory brainstem response OR otoacoustic emissions OR tympanometry OR hearing OR vestibular OR vertigo OR dizziness OR caloric OR tinnitus) OR (S1)  S3: (audiometry OR pure tone audiometry OR pure tone average OR dpoae) OR (S2)  S4: (cogan's syndrome) AND (S3) |
| Cochrane: 0 results (08/01/23) | Cogan AND auditory OR vestibular |

**Appendix B – References of Included Studies**

1. Albrite JP, Resnick DM. Cogan's Syndrome: Case Presentations. Article. *Archives of Otolaryngolog*. 1961;74(5):501-506. doi:10.1001/archotol.1961.00740030512003

2. Azami A, Maleki N, Kalantar Hormozi M, Tavosi Z. Interstitial Keratitis, Vertigo, and Vasculitis: Typical Cogan's Syndrome. *Case Rep Med*. 2014;2014:830831. doi:10.1155/2014/830831

3. Baumann A, Helbling A, Oertle S, Häusler R, Vibert D. Cogan's syndrome: clinical evolution of deafness and vertigo in three patients. *Eur Arch Otorhinolaryngol*. Jan 2005;262(1):45-9. doi:10.1007/s00405-004-0738-8

4. Beccastrini E, Emmi G, Squatrito D, Vannucchi P, Emmi L. Infliximab and Cogan's syndrome. Letter. *Clinical Otolaryngology*. 2010;35(5):441-442. doi:10.1111/j.1749-4486.2010.02180.x

5. Bellucci RJ, Grobeisen B, Sah BC. Bilateral sudden deafness in Cogan's syndrome. *Bull N Y Acad Med*. Jun 1974;50(6):672-81.

6. Benitez JT, Arsenault MD, Licht JM, Cohen SD, Greenberg RV. Evidence of central vestibulo-auditory dysfunction in atypical Cogan's syndrome: a case report. *Am J Otol*. Mar 1990;11(2):131-4.

7. Best C, Thömke F, Hitzler W, Dieterich M. Plasmapheresis as effective treatment in chronic active Cogan-I-syndrome. Letter. *Immunology Letters*. 2013;150(1-2):87-88. doi:10.1016/j.imlet.2012.12.010

8. Bhandari GS, Duggal L, Jain N, Patel J. Cogan syndrome: An autoimmune eye and ear disease with systemic manifestations. *Natl Med J India*. Nov-Dec 2019;32(6):349-351. doi:10.4103/0970-258x.303611

9. Boyd GG. Cogan's syndrome; report of two cases with signs and symptoms suggesting periarteritis nodosa. *AMA Arch Otolaryngol*. Jan 1957;65(1):24-5. doi:10.1001/archotol.1957.03830190026006

10. Bunker DR, Kerr LD. Rituximab Not Effective for Hearing Loss in Cogan's Syndrome. *Case Rep Rheumatol*. 2016;2016:8352893. doi:10.1155/2016/8352893

11. Cabezas-Rodríguez I, Brandy-García A, Rodríguez-Balsera C, Rozas-Reyes P, Fernández-Llana B, Arboleya-Rodríguez L. Late-onset Cogan's syndrome associated with large-vessel vasculitis. *Reumatol Clin (Engl Ed)*. Sep-Oct 2019;15(5):e30-e32. doi:10.1016/j.reuma.2017.05.002

12. Cassis AM. Cochlear Ossification in a Patient with Cogan's Syndrome Undergoing Bilateral Cochlear Implantation. *Case Rep Otolaryngol*. 2018;2018:7395460. doi:10.1155/2018/7395460

13. Cochrane AD, Tatoulis J. Cogan's syndrome with aortitis, aortic regurgitation, and aortic arch vessel stenoses. *Ann Thorac Surg*. Nov 1991;52(5):1166-7. doi:10.1016/0003-4975(91)91304-e

14. Cote DN, Molony TB, Waxman J, Parsa D. Cogan's syndrome manifesting as sudden bilateral deafness: diagnosis and management. *South Med J*. Sep 1993;86(9):1056-60. doi:10.1097/00007611-199309000-00018

15. Cundiff J, Kansal S, Kumar A, Goldstein DA, Tessler HH. Cogan's syndrome: a cause of progressive hearing deafness. *Am J Otolaryngol*. Jan-Feb 2006;27(1):68-70. doi:10.1016/j.amjoto.2005.07.006

16. Dekker JJ, Dinant HJ, Van Soesbergen RM. Cogan's syndrome as an extra-articular manifestation of rheumatoid arthritis. *Clin Rheumatol*. Jul 1996;15(4):374-7. doi:10.1007/bf02230360

17. Del Carpio J, Espinoza LR, Osterland CK. Cogan's syndrome and HLA BW17. *N Engl J Med*. Nov 25 1976;295(22):1262-3. doi:10.1056/nejm197611252952223

18. Djupesland G, Flottorp G, Hansen E, sjaastad O. Cogan syndrome. The audiological picture. *Arch Otolaryngol*. Mar 1974;99(3):218-25. doi:10.1001/archotol.1974.00780030226014

19. Edrees A, Tran J, Thompson G, Watson KR, Godfrey W, Abdou NI. Cogan's syndrome presenting as Sjögren's syndrome followed by acute aortic regurgitation. *Clin Rheumatol*. May 2003;22(2):156. doi:10.1007/s10067-002-0666-1

20. Fidler H, Jones NS. Late onset Cogan's syndrome. *J Laryngol Otol*. May 1989;103(5):512-4. doi:10.1017/s0022215100156749

21. Forli F, Passetti S, Neri E, Gianfelice D, Berrettini S. A rare association between neurofibromatosis type I and Cogan's syndrome: case report. *Audiological Medicine*. 2009;7(4):241-245. doi:10.3109/16513860903076407

22. Fricker M, Baumann A, Wermelinger F, Villiger PM, Helbling A. A novel therapeutic option in Cogan diseases? TNF-alpha blockers. *Rheumatol Int*. Mar 2007;27(5):493-5. doi:10.1007/s00296-006-0252-y

23. Georgakopoulos CD, Makri OE, Exarchou AM, Pharmakakis N. Atypical Cogan's syndrome presenting as bilateral endogenous endophthalmitis. *Clin Exp Optom*. Jan 2014;97(1):87-9. doi:10.1111/j.1444-0938.2012.00787.x

24. Ghadban R, Couret M, Zenone T. Efficacy of infliximab in Cogan's syndrome. *J Rheumatol*. Dec 2008;35(12):2456-8. doi:10.3899/jrheum.080203

25. Gonçalves RM, Curi AL, Campos WR, Oréfice F, Machado DO. Posterior scleritis in Cogan's syndrome. *Ocul Immunol Inflamm*. Jun 2004;12(2):149-52. doi:10.1080/09273940490895362

26. Hafner S, Seufferlein T, Kleger A, Müller M. Aseptic Liver Abscesses as an Exceptional Finding in Cogan’s Syndrome. Article. *Hepatology*. 2021;73(5):2067-2070. doi:10.1002/hep.31547

27. Hara K, Umeda M, Segawa K, et al. Atypical Cogan's Syndrome Mimicking Giant Cell Arteritis Successfully Treated with Early Administration of Tocilizumab. *Intern Med*. Oct 5 2021;doi:10.2169/internalmedicine.7674-21

28. Hirvonen TP, Aalto H. Recovery of bilateral vestibular loss in Cogan's syndrome--a case report. *Otol Neurotol*. Dec 2013;34(9):1736-8. doi:10.1097/MAO.0b013e3182953154

29. Hurelbrink CB, Ell J. Neurological picture. Vestibulocochlear MRI abnormality in a case of Cogan's syndrome. *J Neurol Neurosurg Psychiatry*. Jun 2011;82(6):657-8. doi:10.1136/jnnp.2010.231522

30. Ikeda M, Okazaki H, Minota S. Cogan's syndrome with antineutrophil cytoplasmic autoantibody. *Ann Rheum Dis*. Aug 2002;61(8):761-2. doi:10.1136/ard.61.8.761

31. Im GJ, Jung HH. Side selection for cochlear implantation in a case of Cogan's syndrome. *J Laryngol Otol*. Mar 2008;122(3):310-3. doi:10.1017/s0022215107008742

32. Jung DH, Nadol JB, Jr., Folkerth RD, Merola JF. Histopathology of the Inner Ear in a Case With Recent Onset of Cogan's Syndrome: Evidence for Vasculitis. *Ann Otol Rhinol Laryngol*. Jan 2016;125(1):20-4. doi:10.1177/0003489415595426

33. Kamakura T, Lee DJ, Herrmann BS, Nadol JB, Jr. Histopathology of the Human Inner Ear in the Cogan Syndrome with Cochlear Implantation. *Audiol Neurootol*. 2017;22(2):116-123. doi:10.1159/000477534

34. Karni A, Sadeh M, Blatt I, Goldhammer Y. Cogan's syndrome complicated by lacunar brain infarcts. *J Neurol Neurosurg Psychiatry*. Feb 1991;54(2):169-71. doi:10.1136/jnnp.54.2.169

35. Kawasaki Y, Uehara T, Kawana S. Cutaneous Vasculitis in Cogan's Syndrome: A Report of Two Cases Associated with Chlamydia Infection. *J Nippon Med Sch*. 2018;85(3):172-177. doi:10.1272/jnms.JNMS.2018_85-25

36. Klement V, Hahn A, Hojdarova A, Sejna I. Cogan's syndrome: a case report. *Acta Otolaryngol*. Oct 2007;127(10):1115-7. doi:10.1080/00016480701200301

37. Kondo Y, Ito S, Ohi Y, et al. Atypical Cogan's syndrome with aortitis. *Intern Med*. 2009;48(12):1093-7. doi:10.2169/internalmedicine.48.1917

38. Kougkas N, Bertsias G, Stratoudaki R, Avgoustidis N. Successful treatment of Cogan’s syndrome with tocilizumab. Letter. *Scandinavian Journal of Rheumatology*. 2021;50(4):330-331. doi:10.1080/03009742.2020.1818822

39. Lee SU, Kim JS, Hyon JY, et al. Pearls & Oy-sters: Cogan syndrome: A potentially grave disorder of audiovestibulopathy with many faces. *Neurology*. Jul 2 2019;93(1):39-41. doi:10.1212/wnl.0000000000007733

40. Lepur D, Vranjican Z, Himbele J, Barsić B, Klinar I. Atypical Cogan's syndrome mimicking encephalitis. *Scand J Infect Dis*. 2004;36(6-7):524-7. doi:10.1080/00365540410020253

41. Lima AKSdM, Garcia CAdA, de Faria MAR, Costa Uchoa UB. Cogan's syndrome: Ocular findings in an atypical case. Article. *Arquivos Brasileiros de Oftalmologia*. 2006;69(6):937-940. doi:10.1590/s0004-27492006000600027

42. Maalikjy Akkawi N, Mattioli F, Pezzini A, Vignolo LA. A case of Cogan's syndrome. *Neurol Sci*. Jun 2000;21(3):183-4. doi:10.1007/s100720070095

43. Maikap D, Pradhan A, Padhan P. A Rare Case of Atypical Cogan's Syndrome Presenting as Encephalitis. *Mod Rheumatol Case Rep*. Dec 27 2021;doi:10.1093/mrcr/rxab055

44. Manto MU, Jacquy J. Cerebellar ataxia in Cogan syndrome. *J Neurol Sci*. Mar 1996;136(1-2):189-91. doi:10.1016/0022-510x(95)00340-8

45. Migliori G, Battisti E, Pari M, Vitelli N, Cingolani C. A shifty diagnosis: Cogan's syndrome. A case report and review of the literature. *Acta Otorhinolaryngol Ital*. Apr 2009;29(2):108-13.

46. Miserocchi E, Baltatzis S, Foster CS. A case of atypical Cogan's syndrome with uncommon corneal findings. *Cornea*. Jul 2001;20(5):540-2. doi:10.1097/00003226-200107000-00021

47. Montes S, Rodríguez-Muguruza S, Soria V, Olivé A. Atypical Cogan' syndrome associated with sudden deafness and glucocorticoid response. *Reumatol Clin*. Jul-Aug 2014;10(4):267-8. doi:10.1016/j.reuma.2013.11.005

48. Morinaka S, Takano Y, Tsuboi H, Goto D, Sumida T. Familial HLA-B*52 Vasculitis: Maternal, Atypical Cogan's Syndrome with Takayasu Arteritis-mimicking Aortitis and Filial Takayasu Arteritis. *Intern Med*. Aug 1 2020;59(15):1899-1904. doi:10.2169/internalmedicine.4067-19

49. Ndiaye IC, Rassi SJ, Wiener-Vacher SR. Cochleovestibular impairment in pediatric Cogan's syndrome. *Pediatrics*. Feb 2002;109(2):E38. doi:10.1542/peds.109.2.e38

50. Orsoni JG, Laganà B, Rubino P, Zavota L, Bacciu S, Mora P. Rituximab ameliorated severe hearing loss in Cogan's syndrome: a case report. *Orphanet J Rare Dis*. Jun 16 2010;5:18. doi:10.1186/1750-1172-5-18

51. Peeters GJ, Cremers CW, Pinckers AJ, Hoefnagels WH. Atypical Cogan's syndrome: an autoimmune disease? *Ann Otol Rhinol Laryngol*. Mar-Apr 1986;95(2 Pt 1):173-5. doi:10.1177/000348948609500213

52. Phee J, Kennedy A, Saunders N, Hughes E. An unusual presentation of Roth spots in Cogan's syndrome. Letter. *Canadian Journal of Ophthalmology*. 2017;52(5):e196-e197. doi:10.1016/j.jcjo.2017.02.010

53. Pherwani A, Bansal S, Agrawal S, Gillow T. Cystoid macular oedema in Cogans syndrome-a case report. *Cases J*. Nov 20 2008;1(1):339. doi:10.1186/1757-1626-1-339

54. Pouchot J, Vinceneux P, Bouccara D, Sterkers O, Bodelet B. Methotrexate as a steroid-sparing agent in Cogan's syndrome: comment on the concise communication by Richardson. *Arthritis Rheum*. Sep 1995;38(9):1348-9. doi:10.1002/art.1780380936

55. Queirós J, Maia S, Seca M, Friande A, Araújo M, Meireles A. Atypical Cogan's Syndrome. *Case Rep Ophthalmol Med*. 2013;2013:476527. doi:10.1155/2013/476527

56. Raza K, Karokis D, Kitas GD. Cogan's syndrome with Takayasu's arteritis. *Br J Rheumatol*. Apr 1998;37(4):369-72. doi:10.1093/rheumatology/37.4.369

57. Richardson B. Methotrexate therapy for hearing loss in Cogan's syndrome. *Arthritis Rheum*. Oct 1994;37(10):1559-61. doi:10.1002/art.1780371026

58. Shibuya M, Fujio K, Morita K, Harada H, Kanda H, Yamamoto K. Successful treatment with tocilizumab in a case of Cogan's syndrome complicated with aortitis. *Mod Rheumatol*. May 2013;23(3):577-81. doi:10.1007/s10165-012-0691-0

59. Takashi W, Yasuharu T. Fever of unknown origin, bilateral sensorineural hearing loss with canal paresis and uveitis with iridocyclitis and episcleritis: a case of Cogan's syndrome. *BMJ Case Reports*. 2018;2018:1-2. doi:10.1136/bcr-2018-224535

60. Teece KH, Olund AP. Audiological management of Cogan's syndrome. Review. *Seminars in Hearing*. 2011;32(4):308-320. doi:10.1055/s-0031-1291935

61. Togashi M, Komatsuda A, Masai R, et al. Hypertrophic cranial pachymeningitis in a patient with Cogan's syndrome. *Clin Rheumatol*. Jun 2008;27 Suppl 1:S33-5. doi:10.1007/s10067-008-0841-0

62. Touma Z, Nawwar R, Hadi U, Hourani M, Arayssi T. The use of TNF-alpha blockers in Cogan's syndrome. *Rheumatol Int*. Aug 2007;27(10):995-6. doi:10.1007/s00296-007-0373-y

63. Treviño González JL, Soto-Galindo GA, Moreno Sales R, Morales Del Ángel JA. Sudden sensorineural hearing loss in atypical Cogan's syndrome: A case report. *Ann Med Surg (Lond)*. Jun 2018;30:50-53. doi:10.1016/j.amsu.2018.04.030

64. Van Doornum S, McColl G, Walter M, Jennens I, Bhathal P, Wicks IP. Prolonged prodrome, systemic vasculitis, and deafness in Cogan's syndrome. *Ann Rheum Dis*. Jan 2001;60(1):69-71. doi:10.1136/ard.60.1.69

65. Vasileiadis I, Stratoudaki R, Karakostas E. Complete restoration of auditory impairment in a pediatric case of Cogan's syndrome: report of a rare case with long-term follow-up and literature review. *Int J Pediatr Otorhinolaryngol*. Apr 2012;76(4):601-5. doi:10.1016/j.ijporl.2012.01.017

66. Vishwakarma R, Shawn TJ. Cochlear implant in Cogan's syndrome. *Eur Arch Otorhinolaryngol*. Oct 2007;264(10):1121-4. doi:10.1007/s00405-007-0362-5

67. Watanabe K, Nishimaki T, Yoshida M, et al. Atypical Cogan's syndrome successfully treated with corticosteroids and pulse cyclophosphamide therapy. *Fukushima J Med Sci*. Dec 2000;46(1-2):49-54. doi:10.5387/fms.46.49

68. Wilder-Smith E, Roelcke U. Cogan's syndrome. *J Clin Neuroophthalmol*. Dec 1990;10(4):261-5.

69. Yaginuma A, Sakai T, Kohno H, Mitooka K, Kohzaki K, Tsuneoka H. A case of atypical Cogan's syndrome with posterior scleritis and uveitis. *Jpn J Ophthalmol*. Nov 2009;53(6):659-661. doi:10.1007/s10384-009-0729-9

70. Ying YL, Hirsch BE. Atypical Cogan's syndrome: a case report. *Am J Otolaryngol*. Jul-Aug 2010;31(4):279-82. doi:10.1016/j.amjoto.2009.02.013
